# Endophenotype-Informed Association Analyses for Liver Fat Accumulation and Metabolic Dysfunction in the Fels Longitudinal Study

**DOI:** 10.3390/ijms26104812

**Published:** 2025-05-17

**Authors:** Ariana L. Garza, John Blangero, Miryoung Lee, Cici X. Bauer, Stefan A. Czerwinski, Audrey C. Choh

**Affiliations:** 1 School of Public Health, UT Health Science Center, Brownsville, TX 78520, USA; 2 School of Medicine, South Texas Diabetes and Obesity Institute, University of Texas Rio Grande Valley, Brownsville, TX 78520, USA; 3 School of Public Health, Division of Epidemiology, Human Genetics and Environmental Sciences, UT Health Science Center, Brownsville, TX 78520, USA; miryoung.lee@uth.tmc.edu (M.L.); audrey.c.choh@uth.tmc.edu (A.C.C.); 4 School of Public Health, Division of Biostatistics, UT Health Science Center, Houston, TX 77030, USA; 5 School of Health and Rehabilitation Sciences, College of Medicine, Ohio State University, Columbus, OH 43004, USA

**Keywords:** MASLD, endophenotype, linkage, genotype association, family studies

## Abstract

The identification of causal genomic regions for liver fat accumulation in the context of metabolic dysfunction remains a challenging goal. This study aimed to identify potential endophenotypes for liver fat content and employ them in bivariate linkage searches for pleiotropic genetic regions where targeted association analysis is more likely to reveal significant variants. Multiple metabolic risk and adiposity distribution traits were assessed using the endophenotype ranking value. The top-ranked endophenotypes were then used in a bivariate linkage analysis, paired with liver fat content. Quantitative trait loci (QTLs) identified as significant or suggestive were targeted for measured genotype association analyses. The highest-ranked endophenotypes for liver fat accumulation were insulin resistance (IR), visceral adipose tissue (VAT), and high-density lipoprotein cholesterol (HDL-C). The univariate linkage analysis for liver fat content identified one significant QTL at chromosome 17p13.2 (Logarithm of odds score (LOD) = 2.90, *p* = 1.29 × 10^−4^). The bivariate linkage analysis pairing liver fat with IR and VAT improved the localization of two suggestive QTLs at 13q21.31 (LOD = 2.11, *p* = 9.03 × 10^−4^), and 6q21 (LOD = 2.35, *p* = 5.07 × 10^−4^), respectively. Targeted association analyses within the -1-LOD score regions of these QTLs revealed 17 marginally significant single nucleotide polymorphisms (SNPs) associated with liver fat content or its combination with the selected endophenotypes. The endophenotype-informed linkage analysis successfully identified regions suitable for the targeted association analysis of liver fat content, either alone or in combination with IR or VAT, leading to the discovery of marginally significant variants with potential for future functional studies.

## 1. Introduction

MASLD (metabolic dysfunction-associated steatotic liver disease) represents an updated classification of hepatic steatosis that incorporates metabolic risk factors regardless of alcohol use [1]. The MASLD definition differs from the former exclusionary non-alcoholic fatty liver disease (NAFLD) in that it includes people with steatotic liver disease (SLD) and at least one of the following: 1. body mass index (BMI) classified as overweight, 2. diagnosis of type 2 diabetes mellitus (T2D), or 3. BMI classified as normal weight but with evidence of metabolic dysfunction, i.e., abnormal waist circumference, insulin resistance, dyslipidemia, inflammation, pre-diabetes, or hypertension [1]. A recent study estimated the prevalence of SLD at 42.1% (95% CI 40.3–43.9) of the US adult population, with 99.4% of them meeting the definition of MASLD, [1] which is higher than the previously reported prevalence under the NAFLD definition of 30% of the US adult population [2]. Under this new definition, MASLD has also continued to be the fastest growing etiology of hepatocellular carcinoma (HCC), or liver cancer, worldwide [1].

There is strong evidence to suggest that MASLD aggregates in families. Heritability estimates for hepatic steatosis range from 26 to 27%, [3,4] and several genome-wide association studies (GWAS) have been able to identify causal genomic regions associated with different MASLD endpoints including histological assessments, imaging-based liver fat content, liver enzyme measurements, and the presence of HCC [5]. The replication of these findings, however, and the characterization of the shared genetic risk between liver fat accumulation and features of metabolic dysfunction, has been challenging. Furthermore, understanding the genetic underpinnings of these complex disorders is critical to support the development of therapeutic and preventive strategies that can target both liver disease and metabolic dysfunction.

Barriers to the discovery of novel genetic factors affecting liver fat are in part due to the inherent limitations of GWAS, such as requiring very large sample sizes, and the difficulties of assessing liver fat accurately in population-based samples [6]. One potential approach that has been shown to assist in the localization of causal genomic regions for complex disorders is the use of intermediate quantitative phenotypes, or “endophenotypes” in family-based linkage studies [7]. Endophenotypes were first introduced in the field of psychiatry and defined as measurable components that lie in the causal pathway between the genotype and the disease [8], and should meet the following criteria to be considered as such: (1) be associated with the phenotype of interest but not part of its diagnosis, (2) be heritable, and (3) be genetically correlated to phenotype of interest [9]. An endophenotype ranking value (ERV) can then be used to assess the potential usefulness of an endophenotype based on its heritability and genetic correlation estimates. The highest-ranked endophenotypes can then be employed in linkage analyses for the phenotype of interest, which has been shown to increase the likelihood of detecting associated genomic regions in other studies [7]. The localization of significant and suggestive quantitative trait loci (QTLs) through linkage, in turn, helps narrow the search space for targeted association analyses, which allows for the utilization of a more conservative *p*-value compared to performing a genome-wide association scan, and reduces the time and computational resources required to perform such analyses. This technique has not yet been employed in the localization of genetic regions related to liver fat accumulation in the context of metabolic dysfunction.

In this study, we performed a search for endophenotypes by ranking potential candidate endophenotypes for fatty liver disease using data from 704 non-Hispanic white adults from the Fels Longitudinal Study (FLS). Top-ranked endophenotypes were then employed in bivariate linkage analyses paired with liver fat content, to see if this aided in the localization of significant genomic regions exhibiting pleiotropic effects. Separate, univariate linkage analyses for liver fat content and each of the selected endophenotypes were also performed. Lastly, significant and suggestive QTLs identified through linkage for liver fat content and selected endophenotypes were targeted for both univariate and bivariate measured genotype association (MGA) analyses in the search for significant or marginally significant variants.

## 2. Results

### 2.1. Heritability of Liver Fat Content

The mean ± SE magnetic resonance imaging-proton density fat fraction (MRI-PDFF), or liver fat content, in the study sample was 5.95% ± 0.23 and 29.8% of the participants met criteria for steatosis based on the clinically significant cut-point of 5.56% [10]. The median MRI-PDFF was 3.5%, ranging from 0.7 to 37.3%. MRI-PDFF showed a heritability estimate of 52% (SE = 0.087, *p* < 2 × 10^−10^), indicating strong genetic influence. Crude and adjusted heritability estimates for all the assessed phenotypes are presented in Table 1; all of the assessed phenotypes were significantly heritable except for aspartate aminotransferase (AST).

### 2.2. Potential Endophenotypes

Potential endophenotypes examined are presented in Table 2. Insulin resistance measured by HOMA was the highest-ranked endophenotype. The heritability of this measure was *h*^2^ = 0.44 ± 0.09 (*p* = 1.0 × 10^−7^), which means that 44% of the observed variation of this trait is due to additive genetic factors. A substantial genetic correlation was detected between homeostatic model assessment-insulin resistance (HOMA-IR) and liver fat (*ρ*_G_ = 0.85 ± 0.08), suggesting shared genetic determinants. The second-best-ranked endophenotype is the measure of abdominal visceral fat accumulation: VAT, with a higher heritability (*h*^2^ = 0.67 ± 0.08, *p* = 8.08 × 10^−17^), but lower genetic correlation (*ρ*_G_ = 0.67 ± 0.08) to liver fat content than insulin resistance. The 3rd-ranked endophenotype is fasting insulin; however, this measure is highly correlated with the 1st-ranked endophenotype as it is a component of HOMA. The 4th- and 5th-ranked endophenotypes, respectively, are both cardiovascular disease risk factors: high density lipoprotein cholesterol (HDL-C) and triglycerides (TG), and heritability estimates of these measurements are also significant at *h*^2^ = 0.60 ± 0.08 (*p* = 1.27 × 10^−13^) and *h*^2^ = 0.52 ± 0.09 (*p* = 7.14 × 10^−11^), respectively. To avoid redundancy when choosing which endophenotypes to use for the bivariate linkage analysis of liver fat content risk, we picked the highest-ranked endophenotype within each of the following classes: For 1. measures of glucose homeostasis: HOMA-IR; for 2. adiposity measures: VAT and for 3. Cardiovascular disease risk factors: HDL-C. The endophenotypes for the fourth class: 4. measures of liver function: alanine aminotransferase (ALT) and aspartate aminotransferase (AST), ranked lowest overall for endophenotype potential, so none of these features were employed for the bivariate linkage analysis.

### 2.3. Bivariate Linkage Analyses Using Liver Fat and Top-Ranked Endophenotypes

We conducted a genome-wide search for quantitative trait loci exhibiting pleiotropic activity for MASLD disease risk based on MRI-PDFF and each of the selected endophenotypes: HOMA-IR, VAT, and HDL-C. The highest score for MRI-PDFF (natural log-adjusted) by the univariate linkage analysis was located on chromosome (chr.) 17p13.2 with a significant logarithm of odds (LOD) score of 2.90 and a nominal *p*-value of 1.29 × 10^−4^. Evidence of additional suggestive QTLs influencing the selected endophenotypes was also localized: For HOMA-IR (natural log-adjusted) on chr. 13q12.13 (LOD = 2.49, *p* = 3.50 × 10^−4^) and chr. 19q13.2 (LOD = 2.11, *p* = 9.16 × 10^−4^); and for VAT accumulation (inverse normalized) on chr. 7q31.32 (LOD = 2.41, *p* = 4.27 *×* 10^−4^), Chr. 12q24.33 (LOD = 2.24, *p* = 6.62 × 10^−4^), and Chr. 21q22.2 (LOD = 2.20, *p* = 7.22 × 10^−4^). The endophenotype HDL-C (inverse normalized) showed the highest significant peak on Chr. 12q23.3 (LOD = 3.09, *p* = 8.14 × 10^−5^) and another suggestive peak at Chr. 8q22.1 (LOD = 2.45, *p* = 3.92 × 10^−4^). Significant and suggestive QTL locations found in univariate linkage analysis are summarized in Table 3.

The comparison of bivariate vs. univariate LOD scores is shown in Table 4. A potential improvement in localization occurred when pairing MRI-PDFF (natural log adjusted) with HOMA-IR (natural log-adjusted), which amplified the evidence of a suggestive QTL on Chr. 13q13.1 (LOD = 2.11, *p* = 9.03 × 10^−4^). The LOD scores at the location from the univariate analyses for each of the individual traits were 1.77 for MRI-PDFF and 1.61 for HOMA-IR.

Another suggestive QTL was also located for the bivariate analysis of MRI-PDFF (inverse-normalized) paired with VAT (inverse-normalized) on Chr. 6q22.32 (LOD = 2.35, *p* = 5.07 × 10^−4^). The LOD scores at the location from the univariate analysis were 0.12 for MRI-PDFF and 1.38 for VAT. The bivariate QTLs identified for the pairing of liver fat content with HDL-C did locate one suggestive QTL, but this is at the same location as the highest HDL-C peak, and shows virtually null activity for liver fat, which is indicative that most of the variability at that region is driven by HDL-C alone.

### 2.4. Targeted Association Analyses at Significant and Suggestive Univariate and Bivariate QTLs

The highest univariate peak for liver fat, and the two bivariate peaks showing an improvement in QTL localization for the pairing of liver fat and insulin resistance, and the pairing of liver fat and VAT were targeted for MGA analysis. Specifically, we targeted -1-LOD margin on both sides of the linkage signals. The univariate signal for liver fat at Chr. 17p13.2 comprised a total of 3500 HapMap2 single nucleotide polymorphisms (SNPs), so the Bonferroni corrected significance p-value for this region was set at 1 × 10^−5^. The signal for the bivariate analysis of liver fat and insulin resistance in Chr. 13q31.1 comprised 9500 HapMap2 SNPs, so the Bonferroni corrected significance p-value for this region was set at 5 × 10^−6^. And lastly, the bivariate signal for the analysis of liver fat and VAT in Chr. 6q22.32 comprised 3500 SNPs, so the Bonferroni corrected significance *p*-value was set at 1 × 10^−5^.

No evidence of significant association based on Bonferroni corrected thresholds was observed for liver fat, or each bivariate combination on either of the three locations. However, one variant located at 13q31.1 had a marginal *p*-value of 1.0 × 10^−5^ for liver fat content (rs1571830) and was replicated with a marginal *p*-value of 6.4 × 10^−5^ for the bivariate association with liver fat and insulin resistance. As a sample control, two of the most commonly known variants related to SLD in the patatin-like phospholipase domain-containing 3 gene (PNPLA3), also known as the adiponutrin gene located on Chr. 22q13.31, were also assessed under MGA for liver fat content. These and other variants with marginal evidence of association are summarized in Table 5.

## 3. Discussion

The use of endophenotypes in bivariate linkage analyses in this study facilitated the location of suggestive regions potentially affecting both liver fat deposition and the selected endophenotypes: insulin resistance and VAT, narrowing the search window to perform further targeted association analyses. Of particular interest is the peak located on Chr. 13q31.1. This region contains the *SPRY2* (Sprouty homolog 2) gene which has been found to be associated with T2D by several GWA studies, [11] but not with liver fat accumulation.

The region under the highest LOD for liver fat, located on 17p13.2, on the other hand, contains several genes that have been identified to influence both adiposity distribution and metabolic biomarkers, but not liver fat accumulation directly. For example, the WSC-containing domain (*WSCD1*) gene, located at 17p13.2, which has been found to be associated with HDL-C levels in a Chinese twin-study, [12] and the Rab GTPase-binding effector protein-1 (*RABEP1*) gene that has been found to be associated with lipid and adiposity biomarkers, specifically the following: non-HDL cholesterol, TG and total cholesterol levels, [13] BMI, [14], and BF% [15].

When it comes to the marginally associated HapMap 2 SNPs that were identified through targeted association analyses with liver fat accumulation, and the bivariate effect on liver fat and insulin resistance, or liver fat and VAT, some of these SNPs were found in current genomic databases to be related to some genes that in the literature have been linked to outcomes that could be of interest to the pathways concerned for in MASLD. For example, rs1571830 in Chr. 13 was found to be in linkage disequilibrium with variants in two genetic regions: the long intergenic non-protein coding RNA 351 (*LINC00351*) and the *SLIT* and *NTRK*-Like Family Member 6 gene (*SLITRK6*), which have been implicated in obesity-related biological pathways and traits [16]. For the SNPs located in chromosome 17, 6 out of the 10 (rs218670, rs170149, rs218698, rs184295, rs218697, and rs218695) are coded for in the solute carrier family 13 member 5 gene (*SLC13A5*), which is linked to citrate metabolism and whose function has been implicated in several conditions, such as chronic kidney disease, [17] obesity, insulin resistance, non-alcoholic fatty liver disease, and even cancer [18].

SLC13A5 encodes a sodium-coupled citrate transporter (NaCT), which regulates intracellular citrate availability. Citrate is a key metabolic intermediate in hepatic de novo lipogenesis (DNL), serving both as a substrate and an allosteric regulator for enzymes such as ATP citrate lyase (ACLY). The overactivity of SLC13A5 can elevate hepatic citrate influx, thereby promoting acetyl-CoA production and enhancing lipid biosynthesis, potentially leading to hepatic fat accumulation [19]. Animal models have shown that the overexpression of SLC13A5 in hepatocytes leads to lipid accumulation, while its inhibition confers protection against steatosis [20]. Nevertheless, there is still a scarcity of research specifically examining the regulatory mechanisms behind the expression of SLC13A5 in human liver tissue and its role in MASLD pathogenesis.

Three additional SNPs located in chromosome 17 (rs680625, rs7219134, and rs12150116), were also found to be in linkage disequilibrium with variants in genes: Chromosome 17 open reading frame 100 (C17orf100) and F-box protein 39 (FBX39), which are also both implicated in citrate metabolism [21,22]. Additionally, a marginally significant SNP in chromosome 17 (rs11078484) for both liver fat and insulin resistance is coded for in the Cytochrome B5 Domain-Containing 2 gene (CYB5D2), which has been found to be correlated with diastolic blood pressure (DBP) [23] and T2D [24].

Lastly, there was only one SNP on Chr. 6 that showed a marginally significant association with liver fat content and VAT paired; this SNP is located on an RNA gene: RNF217 antisense RNA 1 (RNF217-AS1), which has been found to be associated with subcutaneous adipose tissue measurements [25]. Five additional rare variants on Chr. 6 (minor allele frequency (MAF) < 0.05) were associated with liver fat content alone; four of them were found to be associated with an uncharacterized gene (LOC105377996), and one additional rare variant was found to be located in the chromosome 6, open reading frame 58 gene: C6orf58, which has been implicated in GWAS with anthropometric traits, such as waist-to-hip adjusted BMI and waist circumference [26].

The use of pedigree-based data signifies an implicit enhancement strategy for finding rare variants by increasing the probability that multiple copies of them exist within the pedigree. This higher volume coupled with targeted strategies such as the use of adequately assessed endophenotypes has been shown by previous studies to facilitate more powerful genetic analyses [7,9], and to our knowledge, this technique had not yet been applied in the search for genetic regions associated with liver fat deposition. Despite the inherent advantages of the methodology applied we do believe that the sample size of our study was a considerable limitation in being able to locate significant variants for liver fat deposition and the related endophenotypes explored.

Based on standard assumptions for additive models, our sample size of 704 participants provided approximately 80% power to detect common variants (MAF ≥ 0.10) with moderate effect sizes (β ≥ 0.30) at a genome-wide threshold of α = 1 × 10^−4^, but was likely underpowered to detect rare or small-effect variants. The application of these techniques to a greater sample size and to any other heritable set of conditions is still promising for being able to locate novel regions of genetic interest. We were, nonetheless, able to identify several marginally significant SNPs under our linkage-identified regions that are implicated in important metabolic processes that may underlie metabolic dysfunction, which highlights the importance of conducting additional, targeted association and functional variant testing in these regions to validate and potentially inform future therapeutic discoveries.

In addition to expanding sample size and replication in diverse populations, future research could also benefit from complementary approaches such as Mendelian randomization (MR) analysis. MR offers a framework for evaluating potential causal relationships between metabolic exposures such as insulin resistance, visceral adiposity, or circulating lipids, with liver fat accumulation, using genetic instruments to reduce confounding. This method has been successfully applied in MASLD research to disentangle directionality and establish etiologic links between metabolic traits and hepatic steatosis [27].

The heritability of liver fat accumulation, as demonstrated in this study, aligns with findings from other cardiometabolic traits such as BMI, insulin resistance, VAT, and lipid profiles, all of which have shown moderate to high heritability in large-scale family and twin studies [28,29,30]. Moreover, several of the loci implicated in our analysis, such as those near SLC13A5, RABEP1, and WSCD1, overlap functionally with pathways previously associated with adiposity, dyslipidemia, and glucose regulation [20,31,32]. These cross-trait consistencies support the relevance and validity of the genetic signals detected here, particularly within shared metabolic networks underpinning liver fat accumulation and related traits.

Although the data collection for this study occurred between 2012 and 2015, the timing of this analysis reflects the increasing availability and maturity of analytical approaches such as MGA within variance component frameworks. These methods have only recently become computationally efficient for large-scale pedigree data. Furthermore, liver fat accumulation continues to be a central phenotype of interest in metabolic disease research, and the genetic contributors identified here remain biologically and clinically relevant. As such, this work contributes timely and foundational insight into the heritable components of hepatic steatosis and its metabolic correlates.

## 4. Materials and Methods

### 4.1. Participants and Study Design

This study was a quantitative genetic analysis (QGA) of data available from the FLS, a research project focusing on the growth, development, and body composition of families initiated in Yellow Springs, Ohio in 1929 (R01HD012252, Czerwinski PD/PI). We included 704 adults from the Fels Longitudinal Study who completed MRI liver fat assessments between 2012 and 2015. Participants with a history of moderate-to-heavy alcohol consumption (greater than 4 drinks on any day for men and greater than 3 drinks on any day for women), were excluded (n = 8), resulting in a total sample size of 704 participants.

All the participants were recruited under institutional review board (IRB) approval at Wright State University in Dayton, Ohio. FLS study participants with no contra-indications for MRI assessments or a self-reported history of chronic liver disease were eligible to participate in this portion of the study. Data analysis for this study was conducted under current IRB approval for UTHealth: HSC-SPH-17-0262 (Lee PI).

### 4.2. Pedigree Structure and Implications

This study involved a total of 704 participants (311 males and 393 females), who were part of 104 families varying in size from 1 to 58 members. The average family size was 7 members, with a median of 3. Table 6 displays the kinships and frequencies of pairwise relationships among the participants in this study.

### 4.3. Data Collection

MRI Assessment of liver fat content: Liver fat was assessed using a Magnetom-Avanto 1.5 Tesla whole-body scanner (Siemens Healthineers, Erlangen, Germany) equipped with the Syngo MR-B15 software [33]. To ensure accurate results, the participants were re-screened for contraindications before MRI assessments following an overnight fasting period of more than 8 h. Motion artifacts were minimized by employing breath-holding techniques during the acquisition of 6 mm axial slices of the liver. The collected scan data were analyzed using the Syngo.via specialized image analysis software: LiverLab (Siemens Healthineers, Erlangen, Germany), [33] operated by a highly skilled analyst with more than 11 years of experience in MRI analysis. Liver fat content was quantified using the modified Dixon method, which measures the proportion of fat-bound protons relative to the total hepatic proton signal [34]. The validity of this modified Dixon MRI method in assessing liver fat content has been extensively compared to proton magnetic resonance spectrometry (H-MRS) assessments, demonstrating a high correlation (Pearson’s r = 0.9936) [34].

MRI Assessment of abdominal adipose tissue: During the liver MRI assessments, measurements of VAT and SAT were also obtained. The detailed protocols for acquiring these measurements have been previously described [35,36]. In summary, depending on the participant, 21 to 40 axial image slices of the abdominal region were combined to calculate individual volumes (cc) of VAT and SAT. The consistency of repeated MRI scans for VAT and SAT measurements is high, with estimated coefficients of variation (CV) of 2.12% and 0.55%, respectively, and intraclass correlation coefficients of 0.9992 and 0.9999, respectively [36].

Body composition: Body composition was evaluated using dual-energy X-ray absorptiometry (DXA) on a Hologic Discovery-A densitometer (Hologic Inc., Marlborough, MA, USA), adhering to the manufacturers’ protocol [37]. The total body scan was analyzed to yield total body measures of bone, fat, and lean tissue. The precision and reliability of DXA are well documented [38,39]. Anthropometric data collected also included weight (kg), stature (cm), and circumferences of the abdomen and hip (cm), following standardized protocols [40].

Metabolic health and cardiovascular risk: These include measurements of fasting glucose (FG, mg/dL), fasting insulin (FI)(mU/L), TG (mg/dL), high-density lipoprotein cholesterol (HDL-C, mg/dL), liver enzymes: ALT, (U/L), and AST (U/L), as indicators of liver health. These were all measured using standard laboratory practices at a commercial laboratory (LabCorp, Burlington, NC, USA). Seated, resting brachial systolic blood pressure (SBP) and diastolic blood pressure (DBP) were obtained through standardized protocols using a mercury sphygmomanometer. Mean arterial pressure (MAP), by means of the formula: MAP = DBP + 1/3(SBP − DBP), [41] was used to combine the systolic and diastolic blood pressure measurements into one variable.

Genomic Data: The study participants had genome-wide SNP data from the Illumina Human 610-Quad BeadChip (Illumina Inc., San Diego, CA, USA), containing more than 550,000 SNPs. SNP loci were checked for Mendelian consistency utilizing SimWalk2 [42], and maximum likelihood techniques that account for pedigree structure were used to estimate allelic frequencies [43]. HapMap2 SNP genotypes were also imputed using MaCH1, [44,45] and also further cleaned using SimWalk2 [42].

### 4.4. Statistical Methods

#### 4.4.1. Quantitative Genetic Analyses

Sequential Oligogenic Linkage Analysis Routines (SOLAR) [46] was used to perform univariate and bivariate quantitative genetic analyses to estimate heritability and genetic correlation estimates between the phenotype for our outcome of interest: MRI-PDFF or liver fat content and each of the phenotypes assessed as potential endophenotypes, while adjusting for age, sex, and their interactions. Quantitative genetic analysis refers to the statistical evaluation of how genetic and environmental factors contribute to variation in continuous traits [47]. Phenotypes that were assessed in both a univariate and a bivariate, pairwise manner, can be grouped in classes and include the following: 1. measures of glucose homeostasis: FG, FI, and HOMA-IR, 2. adiposity measures and distribution: VAT, SAT, BMI, DXA-assessed total body fat percent (BF %), and waist circumference (WC), 3. cardiovascular risk factors: SBP, DBP, MAP, TG, and HDL-C, and 4. measures of liver function: ALT and AST. Inverse normalizations or natural log transformations were applied as deemed appropriate to address non-normality of any of the quantitative trait phenotypes.

#### 4.4.2. Endophenotype Ranking

Using the simplified approach suggested by Glahn, et al. for evaluating potential endophenotypes, [9] candidate traits that were found to be (1) phenotypically associated with (2) significantly heritable and (3) genetically correlated to liver fat accumulation were then ranked against each other using the ERV, an unbiased and empirically derived estimate for the utility of an intermediate phenotype in the pathway for a complex disorder. An advantage of this estimate is that it can be easily derived from the heritability of the quantitative trait of interest (*h_i_*^2^), the square root of the heritability of the potential endophenotype (*h_e_*^2^), and their genetic correlation (*ρ_g_*), as given by the following:

ERV = |√(*h_i_*^2^*)* √*(h_e_*^2^*) ρ_g_|*

ERV estimates range between 0 and 1, with values closer to 1 indicating that the endophenotype and the trait of interest are more likely to be influenced by shared genetic components. The significance of the ERV is assessed by means of a likelihood ratio test comparing the restricted model in which the genetic correlation is set to 0 against the likelihood of another in which the correlation is estimated. Because of this, the corresponding *p*-value is identical to that derived from the assessment of the significance of the genetic correlation.

#### 4.4.3. Bivariate Linkage Analysis

After the ERV had been calculated for all features, a genome-wide linkage search for pleiotropic QTLs influencing both liver fat fraction and the 3 top-ranked endophenotypes was performed, first in a univariate and then in a bivariate manner, to assess whether QTL localization is facilitated by pairing the liver fat fraction trait with each of the top-ranked endophenotypes. Both univariate and bivariate linkage analyses routines have been implemented in SOLAR. Bivariate linkage analysis can improve the detection of shared genetic influences (pleiotropy) by jointly modeling the covariance between two traits, increasing statistical power over univariate models [48]. LOD scores were calculated every 5 centimorgan (cM) across all 23 chromosomes and fine-mapped to every 1 cM in regions where the LOD score was greater than 0.5. LOD scores, which quantify the strength of evidence for linkage, were evaluated against a genome-wide significance threshold derived from the method of Feingold et al. [49], which adjusts for pedigree structure and marker density. Although the classical standard for genome-wide significant linkage is LOD ≥ 3.0, this threshold was derived under specific assumptions of marker independence and fixed pedigree structures. In our study, we applied the Feingold-adjusted threshold of LOD = 2.87, which corresponds to a genome-wide α = 0.05 under the conditions of our linkage scan. Additionally, evidence of suggestive linkage was defined at LOD = 1.67 based on this genome-wide adjusted approach.

#### 4.4.4. Measured Genotype Association Analyses

Single-variant association testing was then conducted under significant or suggestive univariate and bivariate linkage regions by targeting the region spanning -1-LOD score, using MGA analysis in SOLAR. MGA analysis tests the association between specific genetic variants and a trait of interest while accounting for familial relationships through a variance components framework [50]. In this analysis, the interdependence of participants was considered by utilizing the kinship matrix. The kinship matrix quantifies the genetic relatedness among individuals, allowing the model to correct for non-independence due to shared ancestry. Each variant was individually incorporated into the analysis model as a covariate, represented as a genotype dosage of 0, 1, or 2. To determine statistical significance, we used a two-tier thresholding strategy: a genome-wide marginal significance threshold of *p* ≤ 1.0 × 10^−4^ (uncorrected), and a Bonferroni-corrected significance threshold computed based on the number of SNPs analyzed within each linkage-defined region. This allowed us to report both suggestive findings and those meeting more stringent multiple testing correction [51].

## Figures and Tables

**Table 1:** Unadjusted and adjusted heritability estimates for liver disease indicators, glucose homeostasis, adiposity distribution, and cardiovascular disease risk phenotypes.

		Unadjusted Heritability		Adjusted Heritability ^†^	
Phenotype	N	h^2^ ± SE	*p*-Value	h^2^ ± SE	*p*-Value
Steatosis	704	0.598 ± 0.157	2.420 × 10^−5^	0.723 ± 0.171	6.800 × 10^−6^
MRI-PDFF	704	0.445 ± 0.081	3.241 × 10^−9^	0.520 ± 0.087	1.841 × 10^−10^
ALT	623	0.226 ± 0.096	4.982 × 10^−5^	0.253 ± 0.098	2.143 × 10^−3^
AST	623	0.330 ± 0.091	9.100 × 10^−6^	0.274 ± 0.089	1.462 × 10^−4^
FG	670	0.418 ± 0.080	3.826 × 10^−10^	0.424 ± 0.081	1.669 × 10^−10^
FI	688	0.378 ± 0.086	9.000 × 10^−7^	0.377 ± 0.086	1.100 × 10^−6^
HOMA-IR	662	0.449 ± 0.090	4.374 × 10^−8^	0.443 ± 0.090	1.000 × 10^−7^
VAT	704	0.366 ± 0.081	6.000 × 10^−7^	0.665 ± 0.080	8.076 × 10^−17^
SAT	704	0.441 ± 0.075	6.207 × 10^−12^	0.487 ± 0.081	5.721 × 10^−12^
BMI	704	0.493 ± 0.073	8.079 × 10^−14^	0.558 ± 0.076	3.931 × 10^−15^
%BF	676	0.370 ± 0.086	2.000 × 10^−7^	0.493 ± 0.090	1.669 × 10^−9^
WC	704	0.406 ± 0.075	8.875 × 10^−10^	0.520 ± 0.079	8.923 × 10^−13^
SBP	704	0.335 ± 0.080	1.600 × 10^−6^	0.367 ± 0.084	5.000 × 10^−7^
DBP	704	0.304 ± 0.090	1.634 × 10^−4^	0.334 ± 0.093	5.540 × 10^−5^
MAP	704	0.369 ± 0.085	1.500 × 10^−6^	0.373 ± 0.087	2.400 × 10^−6^
TG	693	0.430 ± 0.085	1.524 × 10^−8^	0.519 ± 0.086	7.138 × 10^−11^
HDL-C	693	0.521 ± 0.089	4.802 × 10^−10^	0.603 ± 0.080	1.272 × 10^−13^

^†^ Adjusted for age, sex, and their interactions. Abbreviations: SE, standard error; ALT, alanine aminotransferase; AST, aspartate aminotransferase; VAT, visceral adipose tissue; SAT, subcutaneous adipose tissue; BMI, body mass index; DXA, dual energy x-ray absorptiometry; %BF, percent body fat; SBP, systolic blood pressure; DBP, diastolic blood pressure; MAP, mean arterial pressure; HDL-C, high density lipoprotein cholesterol.

**Table 2:** ERVs for potential endophenotypes in descending order of magnitude by class.

Endophenotype	N	ERV	*p*-Value	*ρ*_g_ ± SE	*h*^2^ ± SE
Glucose homeostasis					
HOMA-IR	662	0.406	1.727 × 10^−8^	0.847 ± 0.078	0.443 ± 0.090
FI	688	0.364	9.568 × 10^−8^	0.822 ± 0.082	0.377 ± 0.086
FG	670	0.282	1.780 × 10^−5^	0.599 ± 0.109	0.424 ± 0.081
Adiposity distribution					
VAT	704	0.392	6.960 × 10^−8^	0.666 ± 0.078	0.665 ± 0.080
WC	704	0.269	1.099 × 10^−3^	0.518 ± 0.085	0.520 ± 0.079
BMI	704	0.267	9.350 × 10^−5^	0.496 ± 0.098	0.558 ± 0.076
DXA %BF	676	0.263	2.054 × 10^−4^	0.521 ± 0.107	0.493 ± 0.090
SAT	704	0.261	1.700 × 10^−4^	0.518 ± 0.104	0.487 ± 0.081
CVD risk factors					
HDL-C	693	0.332	3.480 × 10^−5^	−0.593 ± 0.121	0.603 ± 0.080
TG	693	0.293	4.780 × 10^−5^	0.564 ± 0.100	0.519 ± 0.086
MAP	704	0.187	7.750 × 10^−3^	0.424 ± 0.142	0.373 ± 0.087
SBP	704	0.172	1.148 × 10^−2^	0.393 ± 0.140	0.367 ± 0.084
DBP	704	0.169	1.860 × 10^−2^	0.406 ± 0.158	0.334 ± 0.093
Liver function					
ALT	623	0.163	0.029	0.449 ± 0.185	0.253 ± 0.098
AST	623	0.047	0.508	0.126 ± 0.187	0.274 ± 0.089

All variables are inverse-normalized. Abbreviations: HOMA-IR, homeostatic model assessment-insulin resistance; FI, fasting insulin; FG, fasting glucose; VAT, visceral adipose tissue; WC, waist circumference; BMI, body mass index; BF%, body fat %; SAT, subcutaneous adipose tissue; CVD, cardiovascular disease; HDL-C, high density lipoprotein cholesterol; TG, triglycerides; MAP, mean arterial pressure; SBP, systolic blood pressure; and DBP, diastolic blood pressure.

**Table 3:** Significant and suggestive QTL locations from univariate linkage analyses on liver fat content and selected endophenotypes.

Trait(s)	N	Location (hg.19)	LOD Score	*p*-Value
MRI-PDFF ^†^	696	17p13.2	2.9010 **	1.29 × 10^−4^
HOMA-IR ^†^	656	13q12.13	2.4948 *	3.50 × 10^−4^
		19q13.2	2.1086 *	9.16 × 10^−4^
VAT **^‡^**	696	7q31.32	2.415 *	4.27 × 10^−4^
		12q24.33	2.2385 *	6.62 × 10^−4^
		21q22.2	2.2037 *	7.22 × 10^−4^
HDL-C **^‡^**	685	12q23.3	3.0872 **	8.14 × 10^−5^
		8q22.1	2.4495 *	3.92 × 10^−4^

^†^, log transformed variable; ^‡^, inverse-normalized variable; *, suggestive; and **, significant. Abbreviations: MRI-PDFF, magnetic resonance imaging–proton density fat fraction, i.e., liver fat content; HOMA-IR, homeostatic model assessment–insulin resistance; VAT, visceral adipose tissue; and HDL-C, high-density lipoprotein–cholesterol.

**Table 4:** Suggestive QTL locations from bivariate linkage analyses of liver fat content paired with selected endophenotypes compared to univariate LOD scores.

Location (hg. 19)	N	Trait(s)	LOD Score	Nominal *p*
13q31.1	656	MRI-PDFF ^†^ + HOMA-IR ^†^	2.1144 *	9.03 × 10^−4^
	696	MRI-PDFF ^†^	1.7703 *	2.15 × 10^−3^
	656	HOMA-IR ^†^	1.6081	3.25 × 10^−3^
17p13.2	656	MRI-PDFF ^†^ + HOMA-IR ^†^	2.0901 *	9.60 × 10^−4^
	696	MRI-PDFF ^†^	2.9010 **	1.29 × 10^−4^
	656	HOMA-IR ^†^	0.1527	0.201
6q22.32	696	MRI-PDFF ^‡^ + VAT ^‡^	2.3459 *	5.07 × 10^−4^
	696	MRI-PDFF ^‡^	0.1298	0.220
	696	VAT ^‡^	1.3833	5.80 × 10^−3^
12q23.3	685	MRI-PDFF ^‡^ + HDL-C ^‡^	2.3635 *	4.85 × 10^−4^
	696	MRI-PDFF ^‡^	0.0535	0.310
	685	HDL-C ^‡^	3.0872 **	8.14 × 10^−5^

^†^, log transformed variable; ^‡^, inverse-normalized variable; *, suggestive; and **, significant. Abbreviations: MRI-PDFF, magnetic resonance imaging–proton density fat fraction, i.e., liver fat content; HOMA-IR, homeostatic model assessment–insulin resistance; VAT, visceral adipose tissue; and HDL-C, high-density lipoprotein–cholesterol.

**Table 5:** Marginally associated variants for liver fat content and liver fat paired with selected endophenotypes in targeted MGA analyses.

Variant	Location (hg. 19)	Associated Trait	MAF	*p*-Value
rs738409	22q13.31	MRI-PDFF ^‡^	0.24	1.6 × 10^−4^
rs738408	22q13.31	MRI-PDFF ^‡^	0.24	1.64 × 10^−4^
rs1571830	13q31.1	MRI-PDFF ^‡^, MRI-PDD ^‡ ^+ HOMA-IR ^‡^	0.39	1.0 × 10^−5^, 6.4 × 10^−5^
rs680625	17p13.2	MRI-PDFF ^‡^	0.14	3.1 × 10^−4^
rs7219134	17p13.2	MRI-PDFF ^‡^	0.73	3.65 × 10^−4^
rs12150116	17p13.2	MRI-PDFF ^‡^	0.26	3.67 × 10^−4^
rs218670	17p13.2	MRI-PDFF ^‡ ^+ HOMA-IR ^‡^	0.03	2.4 × 10^−4^
rs170149	17p13.2	MRI-PDFF ^‡ ^+ HOMA-IR ^‡^	0.08	2.7 × 10^−4^
rs218698	17p13.2	MRI-PDFF ^‡ ^+ HOMA-IR ^‡^	0.92	2.7 × 10^−4^
rs184295	17p13.2	MRI-PDFF ^‡ ^+ HOMA-IR ^‡^	0.08	2.9 × 10^−4^
rs218697	17p13.2	MRI-PDFF ^‡ ^+ HOMA-IR ^‡^	0.91	3.1 × 10^−4^
rs218695	17p13.2	MRI-PDFF ^‡ ^+ HOMA-IR ^‡^	0.08	3.2 × 10^−4^
rs11078484	17p13.2	MRI-PDFF ^†^ + HOMA-IR ^†^	0.85	1.1 × 10^−4^
rs781762	6q22.32	MRI-PDFF ^‡ ^+ VAT ^‡^	0.06	1.7 × 10^−4^
rs10080285	6q22.32	MRI-PDFF ^‡^	0.006	2.9 × 10^−4^
rs9491850	6q22.32	MRI-PDFF ^‡^	0.006	3.0 × 10^−4^
rs10085184	6q22.32	MRI-PDFF ^‡^	0.007	3.0 × 10^−4^
rs1080437	6q22.32	MRI-PDFF ^‡^	0.007	4.0 × 10^−4^
rs9491851	6q22.32	MRI-PDFF ^‡^	0.007	4.3 × 10^−4^

Number of individuals with available genotype data = 522, ^†^, log transformed variable; and ^‡^, inverse-normalized variable. Abbreviations: MAF, minor allele frequency; MRI-PDFF, magnetic resonance imaging–proton density fat fraction, i.e., liver fat content; HOMA-IR, homeostatic model assessment–insulin resistance.

**Table 6:** Number and type of pairwise relationships between individuals in the study sample.

Relationship Degree	Relationship Description	N Pairs
1st	Parent–offspring	378
	Siblings	354
2nd	Grandparent–grandchild	65
	Avuncular	450
	Half-siblings	37
	Double 1st cousins	4
3rd	1st cousins and 2nd cousins	12
	Grand avuncular	69
	Half avuncular	63
	1st cousins	500
	Double 1st cousins, 1 removed	19
4th	1st cousins, 1 removed and 2nd cousins, 1 removed	16
	1st cousins, 1 removed	522
	Half 1st cousins	32
	Double 2nd cousins	22
	Double 1st cousins, 2 removed	9
5th and greater	Other	637
	Total relative pairs	3189

## Data Availability

The data presented in this study are available on request from the corresponding author.

## Abbreviations

MASLD	Metabolic dysfunction-associated steatotic liver disease
NAFLD	Non-alcoholic fatty liver disease
BMI	Body mass index
T2D	Type 2 diabetes
HCC	Hepatocellular carcinoma
GWAS	Genome-wide association studies
ERV	Endophenotype ranking value
FLS	Fels Longitudinal Study
MRI	Magnetic resonance imaging
CVD	Cardiovascular disease
MGA	Measured genotype association
QGA	Quantitative genetic analysis
IRB	Institutional Review Board
PDFF	Proton density fat fraction
H-MRS	Proton magnetic resonance spectrometry
VAT	Visceral adipose tissue
SAT	Subcutaneous adipose tissue
CV	Coefficient of variation
DXA	Dual-energy x-ray absorptiometry
FG	Fasting glucose
TG	Triglycerides
HDL-C	High-density lipoprotein cholesterol
ALT	Alanine aminotransferase
AST	Aspartate aminotransferase
SBP	Systolic blood pressure
DBP	Diastolic blood pressure
MAP	Mean arterial pressure
SNP	Single nucleotide polymorphism
SOLAR	Sequential oligogenic linkage analysis routines
FI	Fasting insulin
HOMA-IR	Homeostatic model assessment-estimated insulin resistance
BF%	Body fat percent
WC	Waist circumference
QTL	Quantitative trait loci
LOD	Logarithm of odds
MAF	Minor allele frequency
